# Effects of Precursors and Carbon Nanotubes on Electrochemical Properties of Electrospun Nickel Oxide Nanofibers-Based Supercapacitors

**DOI:** 10.3390/molecules26185656

**Published:** 2021-09-17

**Authors:** Reziwanguli Aihemaitituoheti, Nuha A. Alhebshi, Tuerdimaimaiti Abudula

**Affiliations:** 1 Physics Department, Faculty of Science, King Abdulaziz University, Jeddah 21589, Saudi Arabia; rezwane428@gmail.com; 2 Center of Nanotechnology, King Abdulaziz University, Jeddah 21589, Saudi Arabia

**Keywords:** electrospinning, supercapacitor, nickel oxide, precursor, carbon nanotubes, hybrid electrode

## Abstract

Supercapacitors have been considered as one of the main energy storage devices. Recently, electrospun nanofibers have served as promising supercapacitor electrodes because of their high surface area, high porosity, flexibility, and resistance to aggregation. Here, we investigate the effects of electrospinning parameters and nickel precursors on the nanostructure of electrospun nickel oxide (NiO), as well as on their electrochemical performance as supercapacitor electrodes. In contrast to the case of using nickel nitrate, increasing the nickel acetate molar concentration maintains the flexible fibrous sheet morphology of the as-spun sample during the polycondensation and calcination of NiO. As a result, our flexible electrode of NiO nanofibers derived from nickel acetate (NiO-A) exhibits much better electrochemical performance values than that of nickel nitrate-derived NiO. To further improve the electrochemical storage performance, we combined NiO-A nanofibers with single-walled carbon nanotubes (CNTs) as a hybrid electrode. In both half-cell and full-cell configurations, the hybrid electrode displayed a higher and steadier areal capacitance than the NiO-A nanofibers because of the synergetic effect between the NiO-A nanofibers and CNTs. Altogether, this work demonstrates the potency of the hybrid electrodes combined with the electrospun NiO-A nanofibers and CNTs for supercapacitor applications.

## 1. Introduction

Currently, electrochemical energy storage devices (ESDs) have gained a huge amount of attention worldwide [1]. It has been especially highly desired to create ESDs that can rapidly store and release electric energy with stable cyclic performance, low maintenance cost, and safe operation [2]. Such high-power types of ESDs are called supercapacitors or ultracapacitors, which exhibit higher capacitances than conventional capacitors. The storage mechanisms depend on either ion adsorption and electrical charge on the highly porous electrodes of electric double-layer capacitors (EDLC) or depend on redox reactions between electrodes and electrolytes of Faradic capacitors that are also called pseudocapacitors [3]. One of the key challenges in the industry of sustainable supercapacitors is to develop nanomaterials-based electrodes using cost-effective and non-toxic materials and techniques. Recent advances in nanomaterials science and technology proved that the electrochemical performance of the materials can be remarkably improved by tuning their nanostructure and synthetic conditions [4]. In the past, many types of nanomaterials including nanoparticles, nanorods, nanotubes, thin films, and nanofibers have been utilized for ESDs [4,5,6]. Among them, nanofibers have shown great promise as electrodes because of their large surface area, high porosity, lightweight, flexibility, and resistance to aggregation [3]. Electrospinning is one of the most effective and versatile techniques for producing nanofibers with controllable dimensions [3,7]. This technique allows mixing a wide range of materials including polymers, inorganic materials, and ceramics by initial blending or post-treatment. Moreover, different fiber architectures such as core-shell fibers, hollow fibers, and porous fibers can be achieved by this technique, in addition to producing different patterned fibrous meshes such as twisted sheets and yarns [7,8,9].

The transition metal oxide-based electrodes have obvious reversible redox reactions that lead to larger specific capacitance ranges than carbon-based electrodes for EDLCs [10,11,12]. For example, Dinh et al. [13] developed a micro-sized supercapacitor device using a de-aerated 0.5 M H_2_SO_4_ electrolyte and compared the capacitance performance of hydrous ruthenium oxide (h-RuO_2_) and multi-walled carbon nanotube (MWCNT) within this device. At 10 V/s of scan rate, h-RuO_2_ displayed 3.2 mF cm^−2^ of areal capacitance, while the areal capacitance was only 0.2 mF cm^−2^ for MWCNT under the same condition. Amongst the electrospun nanofibers of transition metal oxides, nickel oxide (NiO) is one of the most widely studied electrodes, owing to its rich natural resources, high electrochemical capacitance, and low toxicity. For instance, Kolathodi et al. [14] fabricated NiO nanofibers with about 15 nm of fiber size by the sol-gel-based electrospinning approach. The nanofibers as a positive electrode exhibited 141 F g^−1^ specific capacitance at 1.5 V of potential in 6 M KOH solution. Ren et al. [15] reported the electrochemical performance of hollow NiO nanofibers modified by citric acid during the electrospinning process. They found that the specific capacitance of the electrode made of the hollow nanofibers was 2.5 times higher than that of solid NiO nanofibers. Kundu et al. [16] designed a binder-free electrode consisted of NiO nanofibers deposited on nickel foam during electrospinning. The designed electrode showed 737 F g^−1^ of high specific capacitance at 2 A g^−1^ and excellent cycling stability.

NiO nanofibers can be synthesized by combining precursors of NiO with high molecular weight polymers in electrospinning, then calcining them at a high temperature (e.g., 500~1000 °C). Some of the most commonly applied precursors of NiO include, but are not limited to, nickel acetate (NiAc) [17,18] and nickel nitrate (NiN) [19,20]. Likewise, polyvinyl alcohol (PVA) [17,20], polyvinylpyrrolidone (PVP) [18], and polyacrylonitrile (PAN) [19] have been extensively used as the support polymers for the NiO nanofibers synthesis by electrospinning. Several studies demonstrated that the concentration of the NiO precursor, polymer concentration, and the polymer/precursor ratio strongly influences the morphological properties of NiO nanofibers and their corresponding electrochemical storage performance [21]. For instance, Khalil et al. [22] synthesized NiO nanofibers via electrospinning a solution of PVA and NiAc. They showed that the morphological structure of NiO nanofibers can be tuned by varying ratios between PVA and NiAc, the concentration of NiAc, and applied voltage. Gazquez et al. [23] fabricated electrospun NiO nanofibers using NiN as a precursor and PVP as a polymer carrier. They suggested that concentrations of NiN and PVP strongly influenced the viscosity and conductivity of the solution, which eventually determined the formation and morphology of the resulting NiO nanofibers. Nevertheless, to the best of our knowledge, the effect of different NiO precursors on the morphological properties of the electrospun NiO nanofibers has not been studied yet. The choice of precursors is critically important in electrospinning, as they strongly influence some of the key electrospinning parameters, such as the solution conductivity, viscosity, and precursor solubility [24,25]. Furthermore, the physical and chemical characteristics of the precursors could also influence the decomposition behavior of the as-spun nanofiber during calcination to produce NiO [25].

Additionally, it has been widely accepted that combining NiO with electronic conductive nanocarbon materials such as carbon nanotubes (CNTs) is an attractive strategy for improving the overall electrochemical capacitance performance of the electrode. The incorporation of CNTs within NiO nanofibers can efficiently prevent their accumulation, significantly enrich their active area for electrochemical reaction, and preserve the stability of the hybrid electrode. Furthermore, the permeation of electrolytes and transference between ions and electrons can be greatly improved through combining NiO with CNTs. Finally, the addition of CNTs can also enlarge the voltage window of NiO for the charging process.

Herein, for the first time, we studied the effects of two different precursors—NiN and NiAc—on the formation, morphology, and electrochemical properties of NiO nanofibers under optimized electrospinning conditions. The nanofibers were characterized by SEM, XRD, and Raman spectra, and their electrochemical storage performance was evaluated in a half-cell electrode testing system. Next, we incorporated CNTs within the NiO nanofibers to further improve their electrochemical capacitance. Finally, we tested the electrochemical properties of the hybrid electrodes in both a half-cell and full-cell configuration.

## 2. Materials and Methods

### 2.1. Nanofiber Preparation

Poly(vinyl alcohol) (PVA, Mw 130000), Nickel(II) acetate tetrahydrate 98% (C_4_H_6_NiO_4_ · 4H_2_O, which is abbreviated below as NiAc), nickel nitrate hexahydrate (Ni(NO_3_)_2_. 6H_2_O, which is abbreviated below as NiN), and single-walled carbon nanotubes (SWCNTs) were purchased from Sigma-Aldrich (St. Louis, MO, USA). Deionized water was used as a solvent. An amount of 0.18 g (6%) of PVA was dispersed in 2.7 mL of cold water under vigorous stirring and stirred at 80 °C for 3 h until complete dissolution. Then, the solution was cooled down, 0.3 mL 1 mol/L of NiN was added into the solution and stirred for 30 min.

Electrospinning was performed in a NANON-01A electrospinning setup (NANON Supply, MECC, Fukuoka, Japan). The solution was placed into a 5 mL syringe and delivered to a 27-gauge blunt metallic needle by Teflon tube. The effects of electrospinning parameters on PVA/NiN nanofibers morphology were studied by changing PVA concentration and applied voltage. After optimizing the electrospinning conditions, NiN and NiAc were electrospun with different concentrations using PVA as polymer support. For NiN, 0.1 and 0.2 mol/L of concentrations were selected, whereas 0.2 and 0.5 mol/L of NiAc were incorporated for the electrospinning. Afterward, the selected electrospun nanofibers were calcined at 650 °C to form NiO.

### 2.2. Characterization Techniques

The morphological characteristics of the prepared fibers were detected using a Field Emission Scanning Electron Microscope (FESEM, JEOL JSM 7600F, Tokyo, Japan). A small part of the fibrous mat was straddling on the specimen stub using a thin layer of conductive carbon tape. To eliminate the charging effect on SEM images, the samples were spin-coated with a conductive thin layer of platinum using Auto Fine Coater (JFC-1600, JEOL JSM 7600F, Tokyo, Japan). The coating was conducted for 30 s at 30 mA and about 3.5 Pa. Afterward, the specimen stub was loaded into the microscopy, and the SEM imaging was performed at 2~5 kV. The size distribution of the nanofibers was calculated by a recently developed digital image processing algorithm. The details of the calculation method are described elsewhere [26].

The chemical structure of the electrospun samples before and after calcination and the related raw materials was analyzed, according to Raman spectra. The spectra were collected on a Raman Microscope (DXR, Thermo Scientific, Waltham, MA, USA) using a 532 nm laser as the excitation source at 8 mW power. An X-ray diffraction system (XRD, ARL X’TRA Thermo Scientific, Waltham, MA, USA) equipped with Cu Ka radiation was used to analyze the crystal structure of the synthesized NiO nanofibers.

### 2.3. Electrochemical Measurements

The electrochemical storage performance of the electrospun samples as supercapacitor electrodes was evaluated by an electrochemical workstation (Model 660E, CH Instruments Incorporation, Bee Cave, TX, USA) using the classical three-electrode testing system, called half-cell. We used platinum wire as counter-electrode and calomel saturated electrode (SCE) of Hg_2_Cl_2_ as a reference electrode in 3 M of KOH electrolyte solution because it is compatible with redox reactions of the Ni-based working electrode [27]. The half-cell was used for optimizing the electrochemical properties of the working electrode with respect to known reference and counter electrodes. When the half-cell provided promising results, the full-cell was constructed using the newly prepared samples as the positive and negative electrodes. The full-cell is called a two-electrode testing configuration. The standard electrochemical experiments were cyclic voltammetry (CV), galvanostatic charge-discharge (CD), and electrochemical impedance spectroscopy (EIS).

From the CD measurements, we calculated the areal capacitance by using the following equation:

C_A_ = IΔt/AΔV, (1)

where (C_A_) is the areal capacitance [F/cm^2^], (I) is the discharge current [A], (Δt) is discharge time [s], (A) is the electrode area [cm^2^], and (ΔV) is the potential drop during discharge in [V] unit [28]. The electrode area in all our experiments was fixed as 1 cm^2^.

The Ni-based sample was mixed with polytetrafluoroethylene (PTFE) binder in a mass ratio of 4:1 and dispersed in ethanol by ultrasonication for 5 min. Then, the mixture was cast on 1 cm^2^ of carbon cloth substrate (Fuel Cell Store, College Station, TX, USA) by drop-by-drop at 60 °C on a hotplate. This conductive substrate is considered to be a flexible current collector to support the electrode. Afterward, the coated substrate was dried in the furnace for 30 min at 60 °C. In parallel, commercial single-walled carbon nanotubes (CNTs) were drop-casted on carbon cloth with PTFE using the same previous steps to fabricate CNTs electrodes. To produce a hybrid electrode, NiO-A, CNTs, and PTFE were mixed in a ratio of 2:2:1 on carbon cloth. In our work, we fabricated half-cells: PVA+N electrode, NiO-N electrode, NiO-A electrode, CNTs electrode, and NiO-A+CNTs hybrid electrode. Further, two full-cell supercapacitors were built up: NiO-A//CNTs device (where NiO-A is the positive electrode and CNTs is the negative electrode) and NiO-A+CNTs//CNTs device (where NiO-A+CNTs is the positive electrode and CNTs is the negative electrode).

In CV experiments, we supplied potential at different scan rates selected from 10 mV s^−1^ to 100 mV s^−1^ and simultaneously measured the produced current. In CD experiments, we executed the areal current selected from 0.06 mA cm^−2^ to 10 mA cm^−2^ and simultaneously measured the charging and discharging potential and time. EIS experiments were performed at a direct current (DC) with 0 V bias and a sinusoidal potential signal of 5 mV amplitude in a frequency range from 0.01 Hz to 100 kHz. EIS data were analyzed using an intricate plane impedance plot called Nyquist plot where the real part of the impedance (Z′) is represented by the *x*-axis and the imaginary part of the impedance (Z″) is assigned at the *y*-axis [29]. The first data point at a high frequency that intercepts with the *x*-axis of the Nyquist plot is correlated with the electronic resistance of the electrode and the current collector substrate in addition to the electrolyte solution, as they are connected in series, so it is called equivalent series resistance (ESR). In the high-frequency region, the diameter of the semicircle corresponds to the charge transfer resistance (R_CT_). It depends on the electrochemical activity of the redox reactions between electrode and electrolyte. The impedance data slop in the low-frequency region is related to the electrolyte ions diffusion into the electrode.

## 3. Results and Discussion

**Figure 1:**
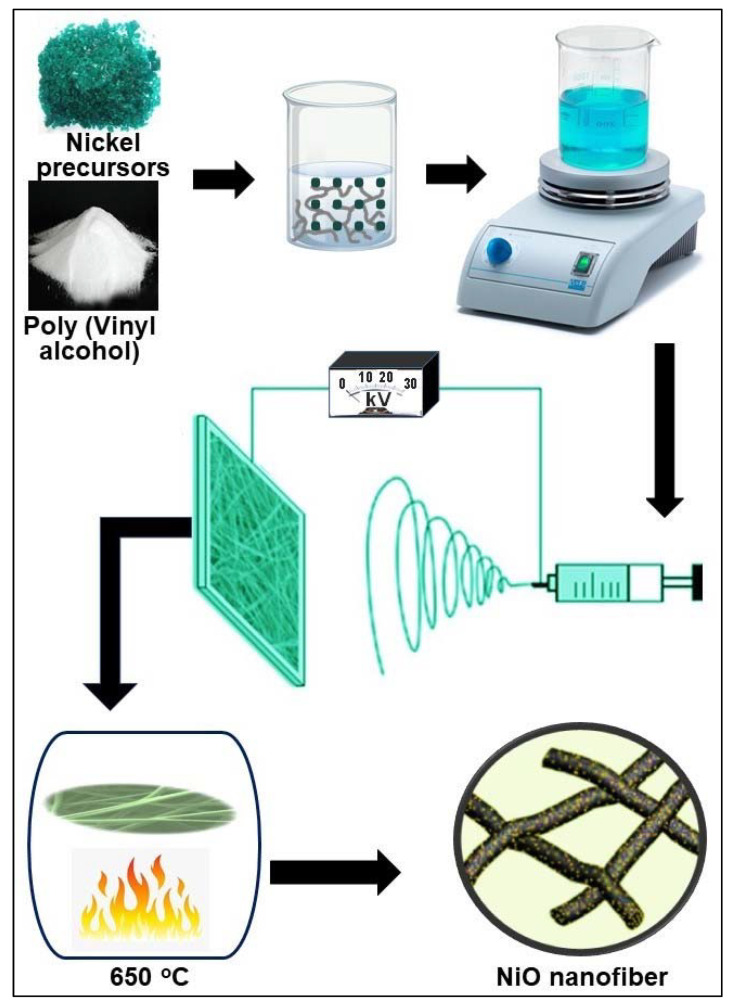
Schematic illustration of NiO nanofibers synthesis using electrospinning.

**Figure 2:**
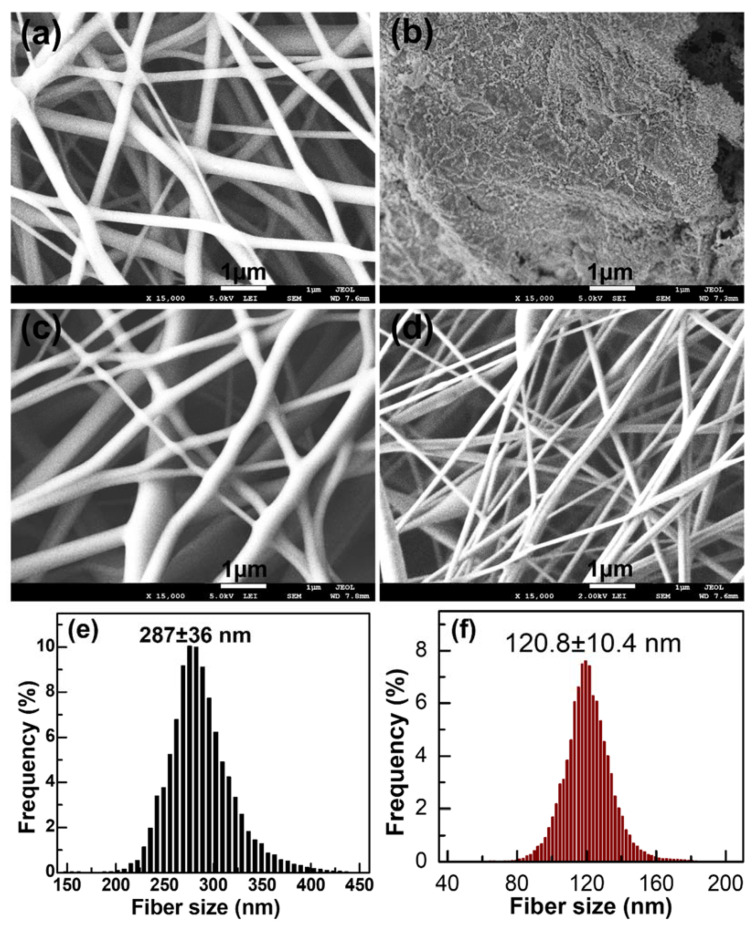
SEM images of (**a**,**b**) NiO-N before and after calcination, respectively; precursor NiN concentration was 0.1 mol/L. (**c**,**d**) NiO-A before and after calcination, respectively; precursor NiAc concentration was 0.5 mol/L, in which the nanofibers were electrospun at 24 kV, PVA concentration was 8% for both samples. (**e**,**f**) Size distribution of NiO-A nanofibers before and after calcination.

The synthesis process of NiO nanofibers by electrospinning of PVA and different NiO precursors is presented schematically in Figure 1. Based on literature findings and considering the chemical properties of PVA [30,31], we initialized our experiments using PVA/NiN solution in water. Afterward, we studied the effect of two detrimental electrospinning parameters: polymer concentration and voltage [26] on the fibers morphology by fixing needle-collector distance at 13 cm, feed rate at 0.3 mL/h, and concentration of NiN at 0.1 mole/L. The morphology of the PVA/NiN nanofibers at different polymer concentrations and different voltages is shown in Figures S1 and S2. We found that in the case of PVA/NiN solution, 24 kV of applied voltage and 8% of PVA concentration yielded smooth and uniform nanofibers without beads or fiber fusion (Figure 2a and Figure S2). This could be because such conditions are critical for the stability and continuity of the Taylor cone so that the balance between the electric field and surface tension can be maintained [32,33]. Additionally, it could provide a suitable flight time for fiber stretching and solvent evaporation, which is favorable for finer fiber formation [34].

Afterward, we applied the calcination technique to create NiO from electrospun PVA/NiN nanofibers. We selected 650 °C as a calcination temperature because it is higher than both the decomposition temperatures of PVA and NiN. Surprisingly, the calcination of the nanofibers resulted mainly in nanoparticles, while occasional ultrathin nanofibers can be observed (Figure 2b). This might be caused by the low concentration of NiO precursor NiN, 0.1 mol/L, which seems insufficient for polycondensation of NiO during calcination [35]. In the second experiment, we increased the concentration of NiN to improve the polycondensation tendency of NiO and produce a particle-free nanofibrous structure. However, when we increased the concentration of NiN to 0.2 mol/L, we observed that there was no stable electrospinning process. Instead of that, there were a lot of droplets that appeared on the collector. We assumed that the ionic conductivity of NiN greatly influenced electrospinning parameters and led the electrospinning to lose its stability. This issue could be solved by using a low conductive nickel precursor, which displays less impact on the stability of optimized electrospinning conditions. We recognized that the ionic conductivity of acetate^−1^ (40.9 × 10^−4^ m^2^ S mol^−1^) is much smaller than the ionic conductivity of NO_3_^−1^ (71.42 × 10^−4^ m^2^ S mol^−1^) [36]. Therefore, we replaced NiN with NiAc as a precursor for proceeding with our next experiments. 

SEM images of the PVA/NiAc nanofibers electrospun from 0.2 mol/L of NiAc, before and after calcination, are illustrated in Figure S3. Unlike NiN, stable electrospinning can be performed with a higher concentration of NiAc, and a clear fiber formation can be observed. After electrospinning, we used 650 °C of calcination temperature to produce NiO nanofibers by decomposing PVA and NiAc, which is the same temperature used for PVA/NiN nanofibers. From the figure, the clear nanofibers’ dominant morphology can be observed after calcination. However, we also noticed that there were few fused fibers and beads in the sample image. This might be because the concentration of NiAc was still not enough for polycondensation of NiO during the calcination as a complete nanofibrous sheet. Therefore, we further increased the precursor NiAc concentration from 0.2 mol/L to 0.5 mol/L (Figure 2c). Eventually, we successfully produced clear nanofibers without any types of fiber fusion after calcination (Figure 2d). We also found that the average fiber size of PVA/NiAc nanofiber decreased from 287 nm to 121 nm after calcination (Figure 2e,f). These measurements, combined with the following results of XRD and Raman, imply that PVA was effectively removed and NiAc successfully decomposed into NiO through calcination [30].

**Figure 3:**
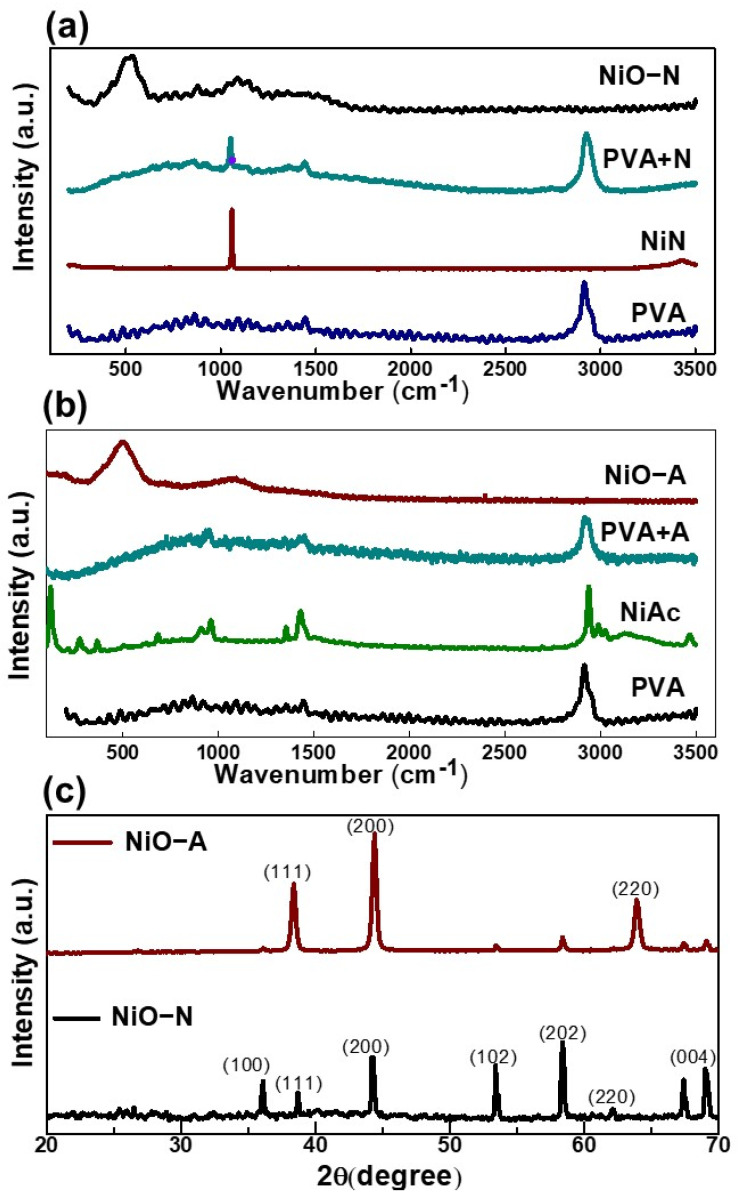
(**a**,**b**) Raman spectra of NiO-N and NiO-A before and after calcination, and their initial components. (**c**) XRD spectra of NiO-N and NiO-A.

The formation of NiO from the PVA/NiN nanofibers was confirmed by Raman spectra (Figure 3a,b). Interestingly, Raman spectra of both PVA and NiN showed single characteristic vibrational bands. Among them, PVA exhibited a strong characteristic band at 2911 cm^−1^, which is assigned to vibration of the aliphatic C-H bond [37]. For NiN, a characteristic band was observed at ~1050 cm^−1^, which corresponded to the symmetric NO_3_^−^ stretch [38]. On the other hand, a strong sharp peak was observed at ~2940 cm^−1^ for NiAc, which corresponded to the C-H stretching vibration [39]. Meanwhile, there were two small peaks at 1428.28 cm^−1^ and 957.31 cm^−1^, which corresponded to the CH_3_ asymmetric bending and (C–C) vibrations, respectively. All these peaks clearly appear in the spectra of electrospun PVA/NiN and PVA/NiAc nanofibers, which implies homogeneous dispersion of PVA and nickel precursors in the nanofibers without deterioration or any chemical reactions. After calcination of the nanofibers, two main characteristic bands appeared at 498 and 1090 cm^−1^, which represented the first and second-order phonon scattering of NiO, respectively [40]. 

The XRD spectra for the NiO resulting from PVA/NiN and PVA/NiAc are compared in Figure 3c. When we prepared NiO by using NiN as the precursor, there were seven characteristic peaks observed at 2θ angles of 36.13°, 38.82°, 44.24°, 53.45°, 58.40°, 62.10°, and 67.45°. These peaks are respectively allocated to diffractions from the (1 0 0), (1 1 1), (2 0 0), (1 0 2), (2 0 2), (2 2 0), and (0 0 4) crystalline planes of the materials, respectively. These values coincided with the diffraction peaks and simultaneously corresponded to the reported NiO (PDF No: 44-1159) [20]. On the other hand, in the case of the NiO generated from the PVA/NiAc nanofibers, there were three main characteristic peaks at 2ϴ values of 38.43°, 44.40°, and 63.91°. Respectively, they were assigned to diffractions from (1 1 1), (2 0 0), and (2 2 0) crystalline planes. The three main diffraction peaks corresponded to the standard face-centered cubic (fcc) NiO (JCPDS No: 04-0835) [41]. This result suggests that NiO resulted from PVA/NiAc nanofibers has a defined atomic structure and much better crystalline phase as compared with NiO produced from PVA/NiN.

**Figure 4:**
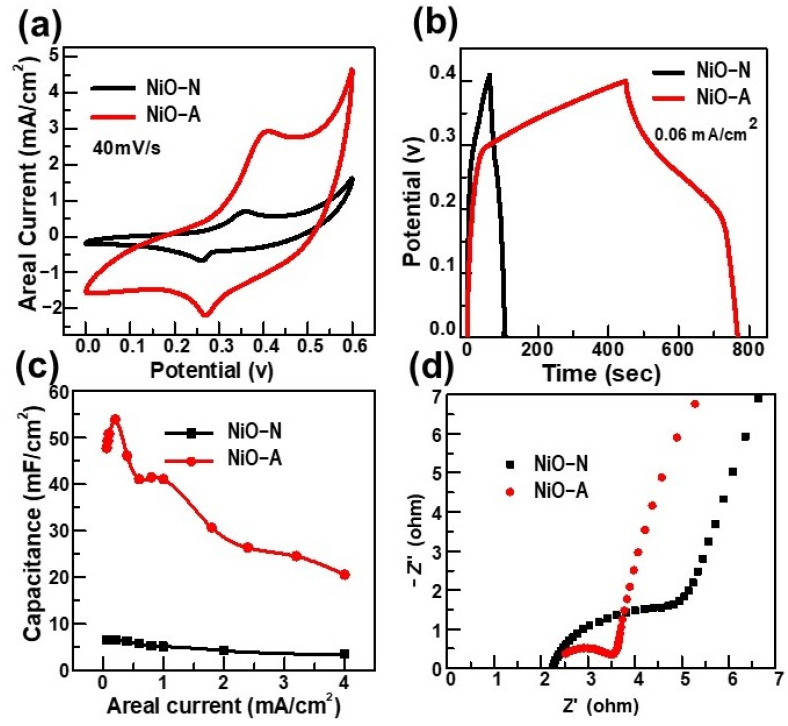
Comparisons of electrochemical storage performance of NiO-N and NiO-A half-cells: (**a**) CV curves; (**b**) CD curves; (**c**) Areal capacitance functions; (**d**) Nyquist plot of EIS.

The electrochemical performance of NiO-based electrodes was evaluated and compared in terms of CV, CD, and EIS. According to Figure 4a, we found that the NiO-A electrode produces a significantly higher areal current than that of the NiO-N electrode in the same potential window at the same scan rate of 40 mV/s. Additionally, the discharge time of the electrode prepared by the NiO-A nanofibers was approximately 7 times longer than that of NiO-N nanoparticles at the same areal current of 0.06 mA/cm^2^ (Figure 4b). As a result, the areal capacitance of NiO-A nanofibers was also found to be 7~9 times higher than the electrode made by NiO-N nanoparticles in all their range of areal current (Figure 4c). The detailed CV and CD plots of NiO-N and NiO-A can be found in Figures S4 and S5. Furthermore, the NiO-A nanofibers as an electrode possessed much smaller transfer resistance compared with the NiO-N nanoparticles, as shown in Nyquist plots of Figure 4d, which confirms that the redox reaction in NiO-A is faster than NiO-N. In the low-frequency region, the straight line of NiO-A makes a larger angle with the *x*-axis than that of NiO-N (~80° vs. ~70°) and has smaller real impedance values than that of NiO-N (4–5 ohm vs. 5–6 ohm). These differences indicate that the ion diffusion and the drift current in NiO-A are faster on the interface of the NiO-A electrode-KOH electrolyte. In general, larger angles than 45° and small impedance are expected for supercapacitors because the electrical charge storage mechanism that occurs at the electrode-electrolyte interface is faster than that which occurs in the entire bulk electrode of batteries [42]. 

Overall, our comparative result suggests that the structural and morphological features of NiO greatly influence its electrochemical storage performance. NiO resulting from nickel acetate possesses numerous advantages, including nanofibrous morphology, defined crystalline phase, high electrochemically active surface area, good electrical conductivity, and resistance to aggregation and/or agglomeration. All these properties are greatly beneficial for the efficiency of the redox reaction occurring between the electrode and the electrolyte. On the other hand, we notice that both the areal capacitances of both NiO-A and NiO-A drop if their charging process was quick (in few seconds) by applying high areal currents (such as 4 mA/cm^2^). It is well known that the chemical redox reactions, in general, may require a longer time than the formation time of the electrical double layer. Therefore, we hybridized NiO with CNTs into a hybrid electrode that could maintain the high capacitance in both slow and fast charging conditions at low and high applied currents, respectively.

**Figure 5:**
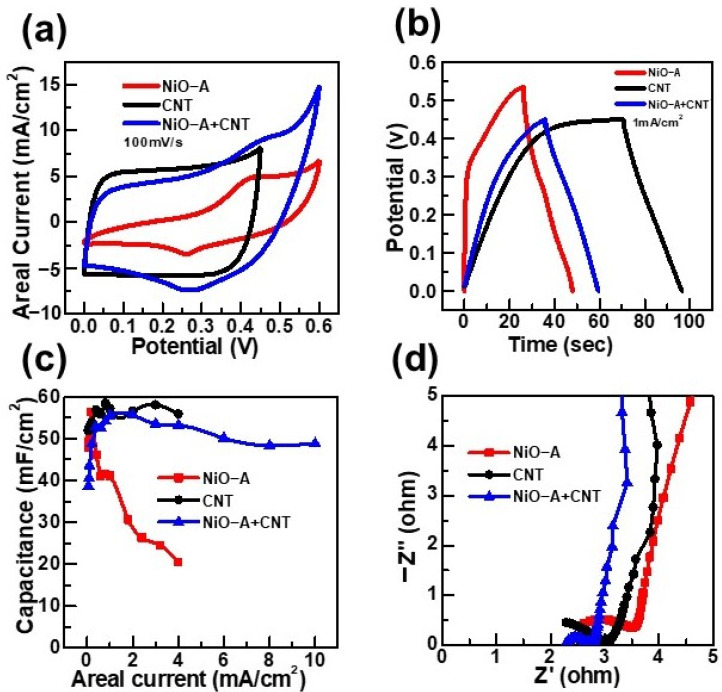
Comparisons of electrochemical storage performance of NiO-A, CNT, and NiO-A+CNT half-cells: (**a**) CV curves; (**b**) CD curves; (**c**) Areal capacitance functions; (**d**) Nyquist plot of EIS.

The electrochemical storage properties of NiO-A, CNTs, and NiO-A+CNTs as working electrodes in half-cells are compared in Figure 5. As shown in Figure 5a, the hybrid electrode combined of NiO-A nanofibers with CNTs results in a significantly broader CV curve than that of individual NiO-A nanofibers or CNTs at 100 mV/s of the same scan rate. Additionally, the hybrid electrode also exhibited significantly higher areal current than the individual electrodes, especially in the redox potential range of 0.4~0.6 V. For instance, the areal current of the hybrid electrode is 9.1 mA/cm^2^ at 0.45 V, while 5.1 and 8.1 mA/cm^2^ of areal currents are obtained at the same potential in cases of the NiO-A nanofibers and CNTs, respectively. It can be also noted that the maximum positive potential that can be used for CNTs is about 0.45 V. While in the case of the hybrid electrode, we were able to extend the potential up to 0.6 V, which is similar to the NiO-A nanofibers and without any indications of oxygen gas evolution as a side reaction. The CD curves in Figure 5b show that by applying a constant current of 1 mA/cm^2^, the potential of the CNT electrode was increased until 0.45 V, then it had spontaneously become a horizontal straight line that was constant with the charging time. The variation of the anodic potential limits can also be observed by different applied areal currents for the same samples (Figures S5a,b, S6e, and S7a,b), such as reaching the potential limit of 0.6 V at 4 mA/cm^2^ for the NiO-A electrode. The NiO-A nanofibers as an electrode at 1 mA/cm^2^ delivered the shortest charging-discharging time and the highest potential limit amongst the three electrodes; all of these factors are correlated with capacitance. The maximum areal capacitances (in F/cm^2^ unit) of NiO-A, CNT, and NiO-A+CNT electrodes are close to each other, but we found that the hybrid electrode has a more stable areal capacitance in a wider range of areal currents than both the NiO-A nanofibers and CNTs (Figure 5c). Taking into account the mass loading on each electrode, the corresponding maximum specific (gravimetric) capacitances (in F/g unit) are also calculated and listed in Table S1. Nevertheless, for thin films on textile substrates, such as carbon cloth, it is recommended that the capacitance should be normalized by footprint area [43,44] because using ultra-small mass loading would lead to overestimated specific capacitances. Moreover, Figure 5d highlights that the hybrid electrode has much smaller R_CT_ compared with the NiO-A nanofibers and CNTs, and smaller ESR than NiO-A. The detailed electrochemical performance of CNTs, in positive and negative potential windows, and the hybrid electrode are described individually in Figures S6 and S7.

Collectively, the comparative result suggests that the hybrid electrode shows superior electrochemical storage performance than both the NiO-A nanofibers and CNTs. This is because the storage mechanisms of the electrode can be driven by both reversible redox reactions and electrical double layers when we combine the NiO-A nanofibers with CNTs. Most importantly, the hybrid electrode owns excellent stable areal capacitances at a wide current range with small ERS and R_CT_ values owing, as anticipated, to the synergetic effect between the NiO-A nanofibers and CNTs [45,46]. In other words, the electrical conductivity, power, and CD rate of the NiO-based electrodes can be enhanced by inserting the CNTs [3]. In parallel, the NiO-A nanofibers could improve the capacitance and the energy of CNTs hybrid electrodes [3,47].

**Figure 6:**
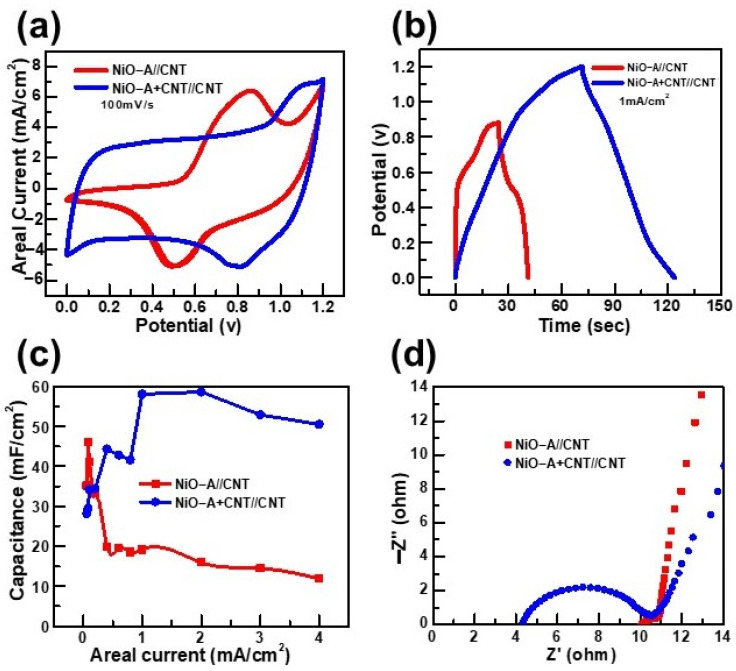
Comparison of electrochemical storage performance of NiO-A//CNT and NiO-A+CNT//CNT as two full-cells: (**a**) CV curves; (**b**) CD curves; (**c**) Areal capacitance functions; (**d**) Nyquist plot of EIS.

Based on the promising results of the half-cells, the electrochemical storage performances of our samples were further evaluated in full-cells to approach the practical prototypes of the supercapacitors. Two asymmetric supercapacitors were constructed: The first one is NiO-A//CNTs, while the second one is NiO-A+CNTs//CNTs. In both devices, the CNTs electrode was fixed as the negative electrode because of its excellent capacitance in the negative postnatal range, as shown in Figure S6b–d. The detailed supercapacitor performance of these full-cells can be found in Figures S8 and S9. Accordingly, we compared the asymmetric supercapacitor’s performance of NiO-A//CNTs and NiO-A+CNTs//CNTs full-cell devices. As shown in Figure 6a, the NiO-A+CNTs//CNTs device results in a significantly broader CV curve with a higher areal current than that of the NiO-A//CNTs device at 100 mV/s, both are in the same potential range of 0~1.2 V. Similarly, the CD potential and time of the NiO-A+CNTs//CNTs are higher than that of the NiO-A//CNTs, both are measured at 1 mA/cm^2^ (Figure 6b). Consequently, we found that the NiO-A+CNTs//CNTs have high and more stable areal capacitances at high areal current (Figure 6c). Moreover, Figure 6d highlights that the NiO-A+CNTs//CNTs have a much smaller ESR compared with the NiO-A//CNTs but larger R_CT_. This can be explained by the addition of CNTs to NiO on the same electrode, which can facilitate the charges to electrically move through the electrode and the substrate but can impede the electrochemical electrons transfer between electrode and electrolyte ions, as the CNTs possess no redox reactions. Altogether, the NiO-A+CNTs//CNTs device showed superior electrochemical storage performance because of the synergetic effects between NiO-A nanofibers and CNTs and excellent performance of CNTs as the negative electrode. Moreover, many studies suggest that asymmetric full-cell configuration provides a wider potential range, higher areal capacitance, and lower series resistance than the symmetric supercapacitors [3,46,48,49].

## 4. Conclusions

In this paper, we studied the effects of nickel precursors and carbon nanotubes incorporation within NiO-based nanofibrous electrodes on their electrochemical storage properties. First, we optimized the electrospinning parameters, and 8 wt% of PVA concentration, 24 kV of applied voltage, 0.3 mL/h of feed rate, and 13 cm of a needle-collector distance were found to be optimum electrospinning parameters to produce uniform nanofibers without fusion from PVA and nickel precursors. Next, we found that contrary to NiN, desirably high concentrations (e.g., 0.5 mol/L) of NiAc can be loaded within PVA solution to perform stable electrospinning. Consequently, we obtained smooth and uniform NiO nanofibers without beads or fusion after calcining the electrospun PVA/NiAc nanofibers. Compared with NiO-N, NiO-A nanofibers exhibited higher areal capacitance, longer CD time, and lower transfer resistance in a half-cell configuration. Next, we combined NiO-A nanofibers with CNTs to make a hybrid electrode. In both a half-cell and full-cell configuration, the hybrid electrode displayed a higher areal capacitance than NiO-A nanofibers. Moreover, the hybrid electrode also exhibited steady areal capacitance values at different current ranges with small transfer resistance. Overall, the hybrid electrode combined with the NiO-A nanofibers and CNTs could be one of the promising electrodes for the supercapacitor. 

## Data Availability

Not applicable.

## Supplementary Materials

The following are available online. Figure S1: SEM images of electrospun PVA+N before calcination, in which nanofibers were electrospun at different polymer concentrations of: (a,b) 6%; (c,d) 7%; (e,f) 8%, Figure S2: SEM images of electrospun PVA+N before calcination, in which nanofibers were electrospun at different applied voltages of: (a,b) 27 kV; (c,d) 25 KV; (e,f) 24 KV; (g,h) 23 KV, Figure S3: SEM images of: (a,b) PVA+A before calcination; (c,d) NiO-A after calcination, in which the nanofibers were electrospun at 24 kV, PVA concentration was 8%, and precursor NiAc concentration was 0.2 mol/L, Figure S4: Electrochemical storage performance of NiO-N nanofibers: (a) CV curves; (b) CD curves; (c) Areal capacitance function; (d) Nyquist plot of EIS, Figure S5: Electrochemical storage performance of NiO-A nanofibers: (a,b) CD curves; (c) Areal capacitance function; (d) Nyquist plot of EIS; (e) CV curves, Figure S6: Electrochemical storage performance of CNTs: (a,b) CV curves in positive and negative potential ranges; (c–e) CD curves in positive and negative potential ranges, Figure S7: Electrochemical storage performance of NiO-A+CNT nanofibers as a hybrid electrode: (a,b) CD curves; (c) Areal capacitance function; (d) CV curves; (e) Nyquist plot of EIS, Figure S8: Electrochemical storage performance of NiO-A//CNTs as an asymmetric full-cell: (a) CV curves; (b,c) CD curves; (d) Areal capacitance function; (e) Nyquist plot of EIS, Figure S9: Electrochemical storage performance of NiO-A+CNTs//CNTs as an asymmetric full-cell: (a,b) CD curves; (c) Areal capacitance function; (d) Nyquist plot of EIS; (e) CV curves, Table S1: The maximum areal capacitance and the corresponding specific capacitance of all our half-cell electrodes.
