# Supplementary material for: Effects of Precursors and Carbon Nanotubes on Electrochemical Properties of Electrospun Nickel Oxide Nanofibers-Based Supercapacitors
Source: Molecules. 2021 Sep 17;26(18):5656. doi: 10.3390/molecules26185656 (PMC8465373; doi:10.3390/molecules26185656)
Supplement: Supplementary file 1 [file molecules-26-05656-s001.zip › molecules-1320731-supplementary.pdf]

Supplimentary Materials

Effects of Precursors and Carbon Nanotubes on Electrochemical Properties of Electrospun Nickel Oxide Nanofibers-Based Supercapacitors

Reziwanguli Aihemaitituoheti [1], Nuha A. Alhebshi [1,*], and Tuerdimaimaiti Abudula [2,*]

[1] Physics Department, Faculty of Science, King Abdulaziz University, Jeddah 21589, Saudi Arabia; rezwane428@gmail.com, nalhebshi@kau.edu.sa
[2] Center of Nanotechnology, King Abdulaziz University, Jeddah 21589, Saudi Arabia; 1202908@gmail.com
* Correspondence: nalhebshi@kau.edu.sa (N.A); 1202908@gmail.com (T.A)

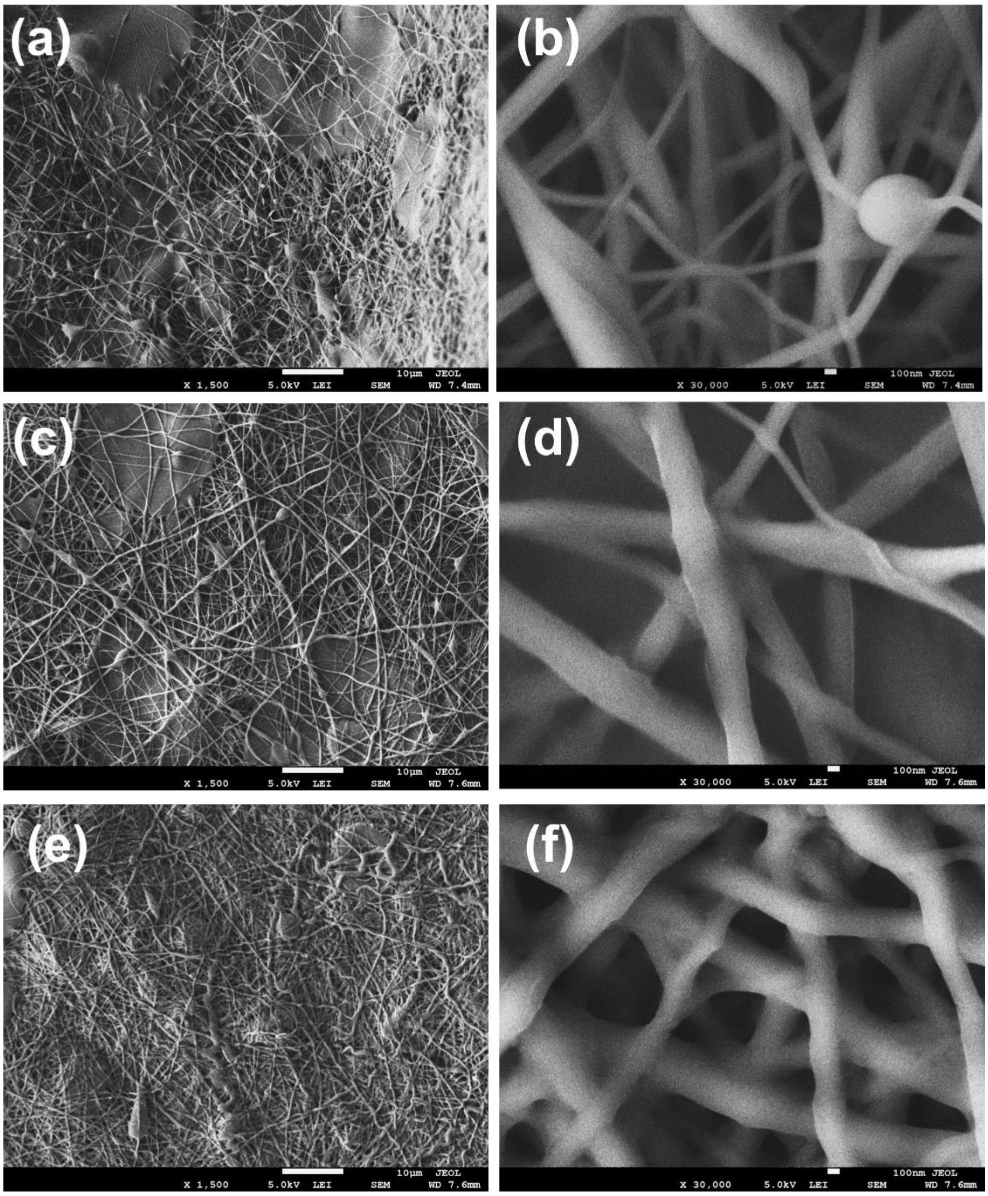

Figure S1. SEM images of electrospun PVA+N before calcination, in which nanofibers were electrospun at different polymer concentrations of: (a, b) 6%, (c, d) 7%, (e, f) 8%.

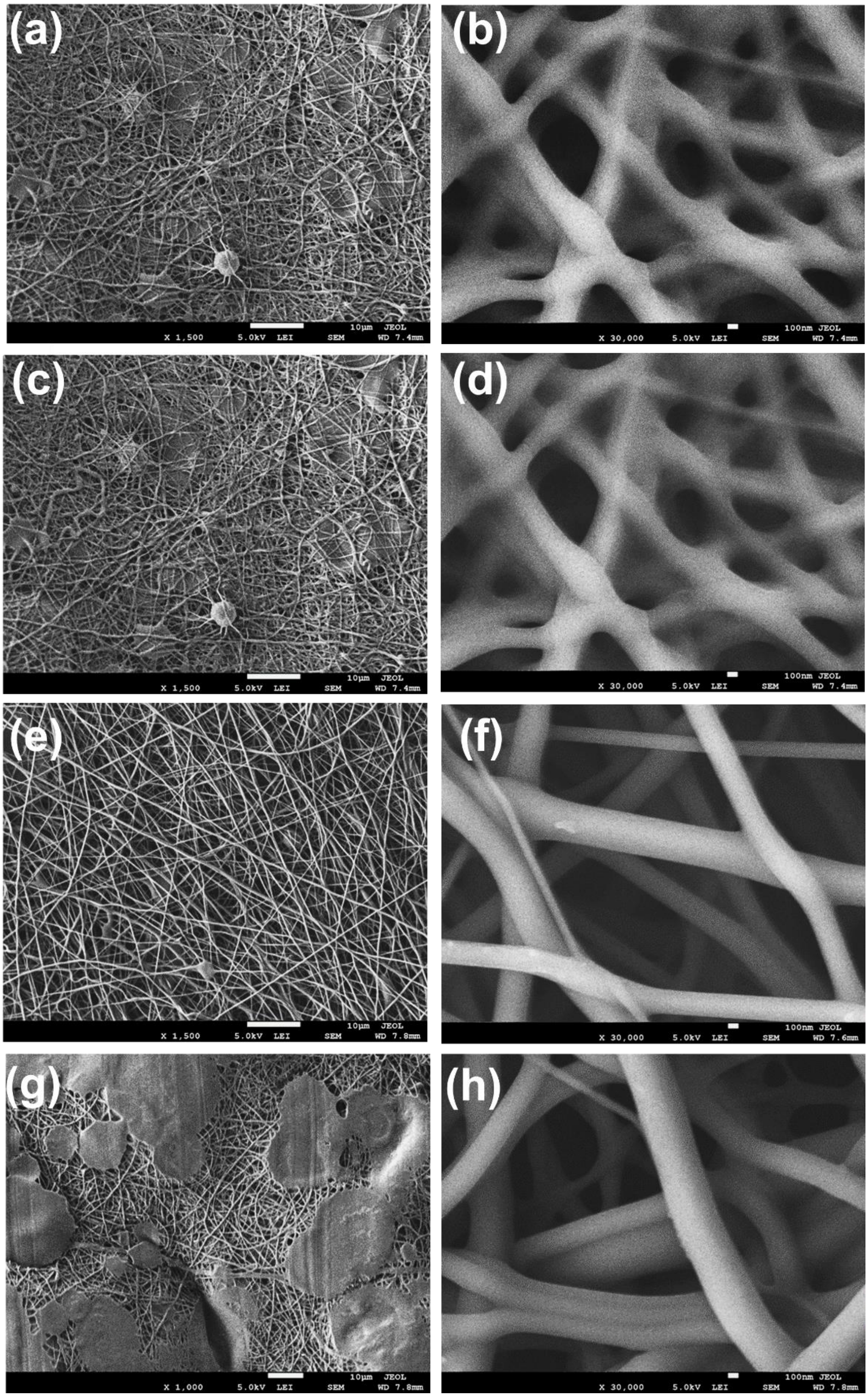

Figure S2. SEM images of electrospun PVA+N before calcination, in which nanofibers were electrospun at different applied voltages of: (a,b) 27 kV, (c, d) 25 KV, (e, f) 24 KV, (g, h) 23 KV.

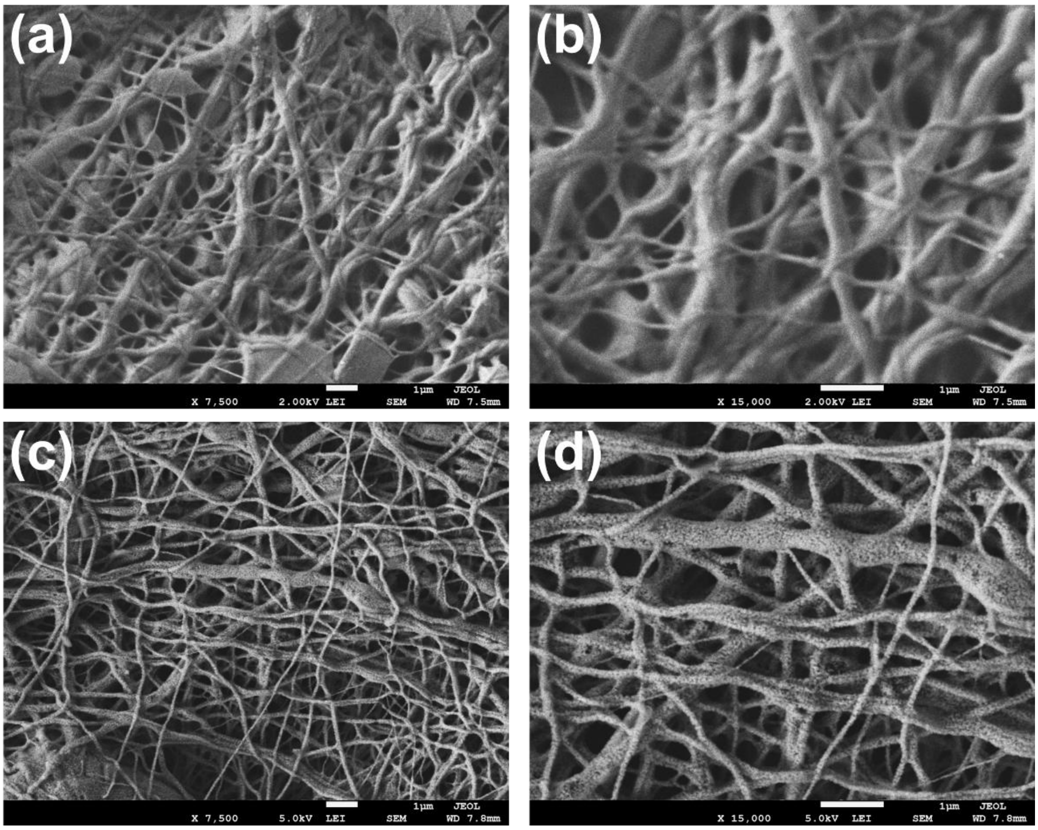

Figure S3. SEM images of: (a,b) PVA+A before calcination, (c,d) NiO-A after calcination, in which the nanofibers were electrospun at 24 kV, PVA concentration was 8% and precursor NiAc concentration was 0.2 mol/L.

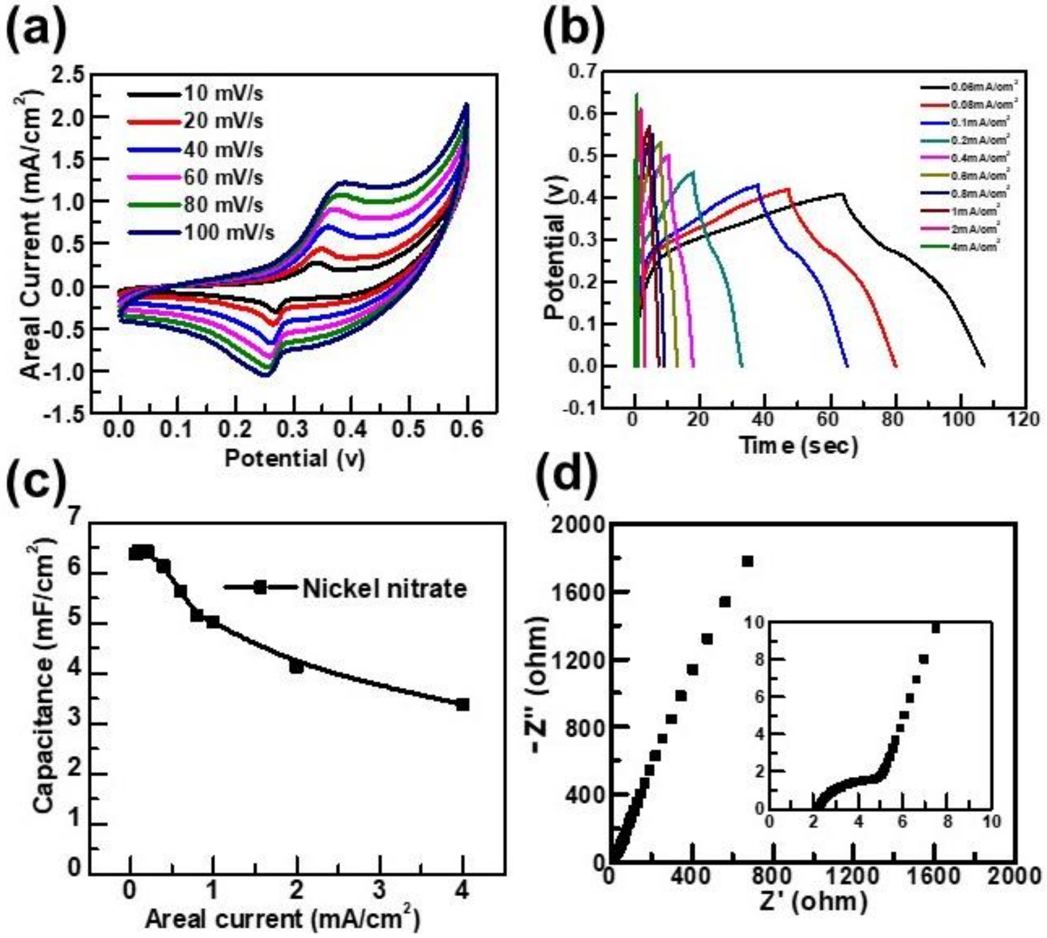

Figure S4. Electrochemical storage performance of NiO-N nanofibers: (a) CV curves, (b) CD curves, (c) Areal capacitance function, (d) Nyquist plot of EIS.

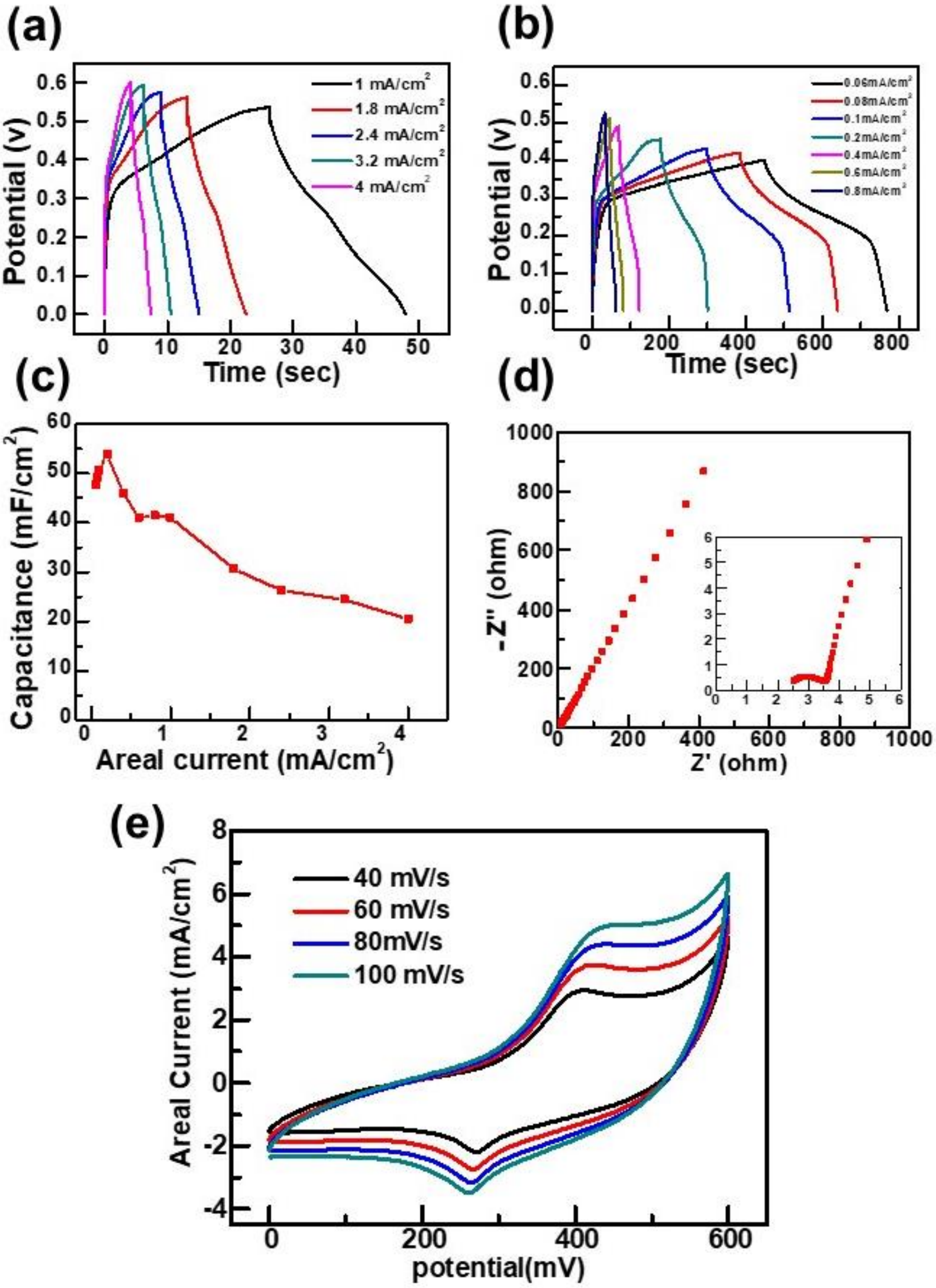

Figure S5. Electrochemical storage performance of NiO-A nanofibers: (a, b) CD curves, (c) Areal capacitance function, (d) Niquist plot of EIS, (e) CV curves.

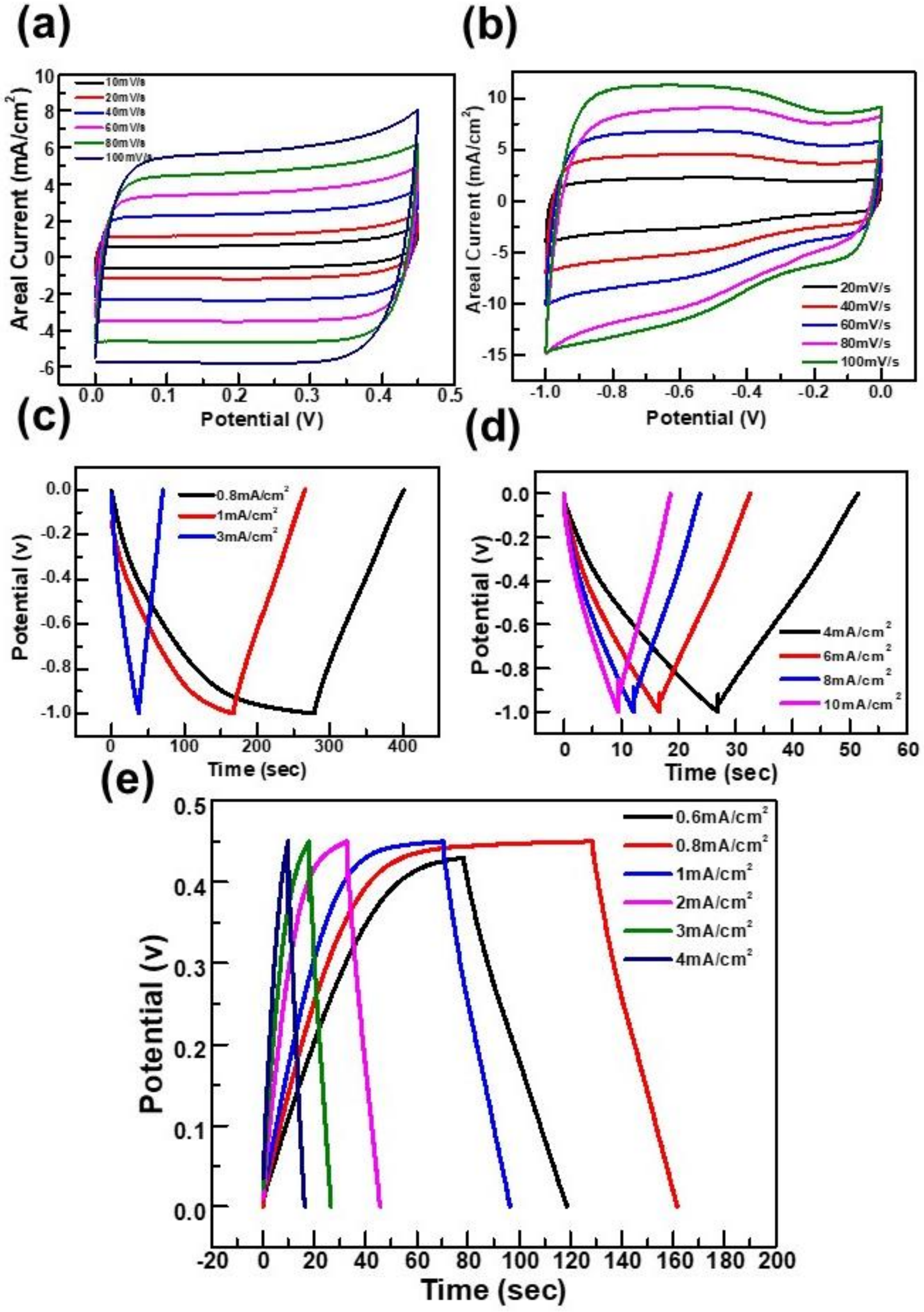

Figure S6. Electrochemical storage performance of CNTs: (a,b) CV curves in positive and negative potential ranges, (c-e) CD curves in positive and negative potential ranges.

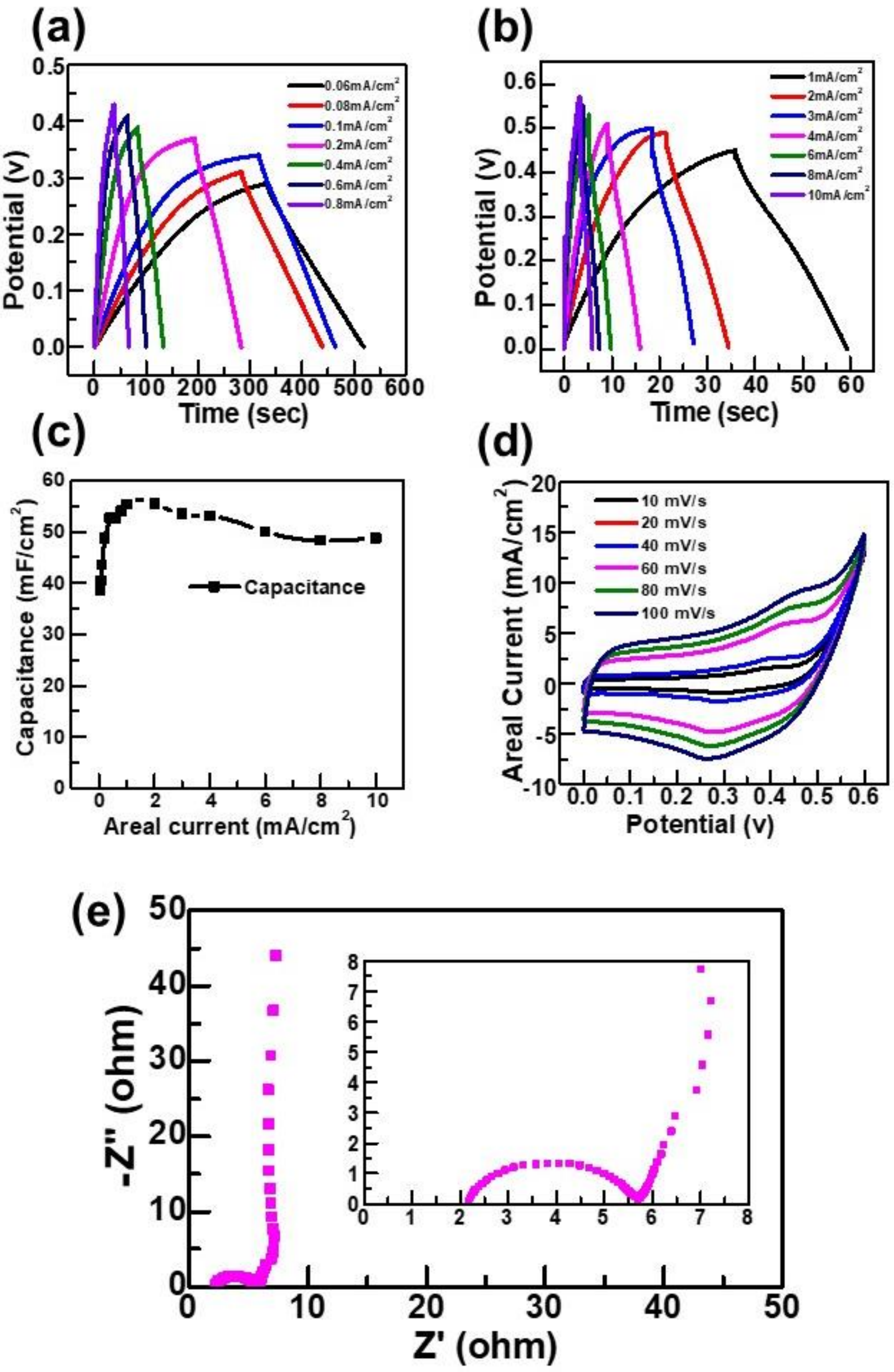

Figure S7. Electrochemical storage performance of NiO-A+ CNT nanofibers as a hybrid electrode: (a, b) CD curves, (c) Areal capacitance function (d) CV curves (e) Nyquist plot of EIS.

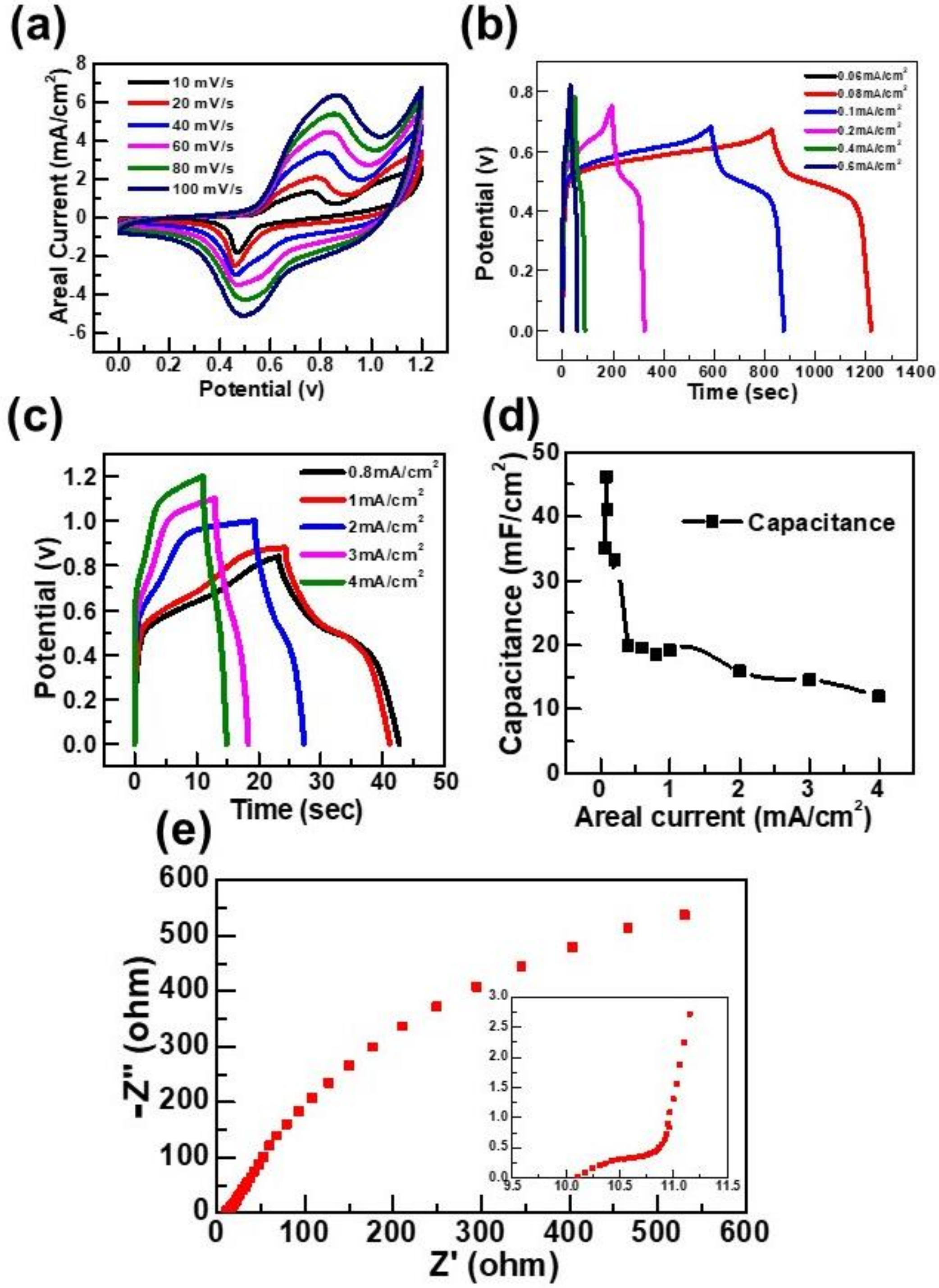

Figure S8. Electrochemical storage performance of NiO-A//CNTs as an asymmetric full-cell: (a) CV curves, (b,c) CD curves, (d) Areal capacitance function, (e) Niquist plot of EIS.

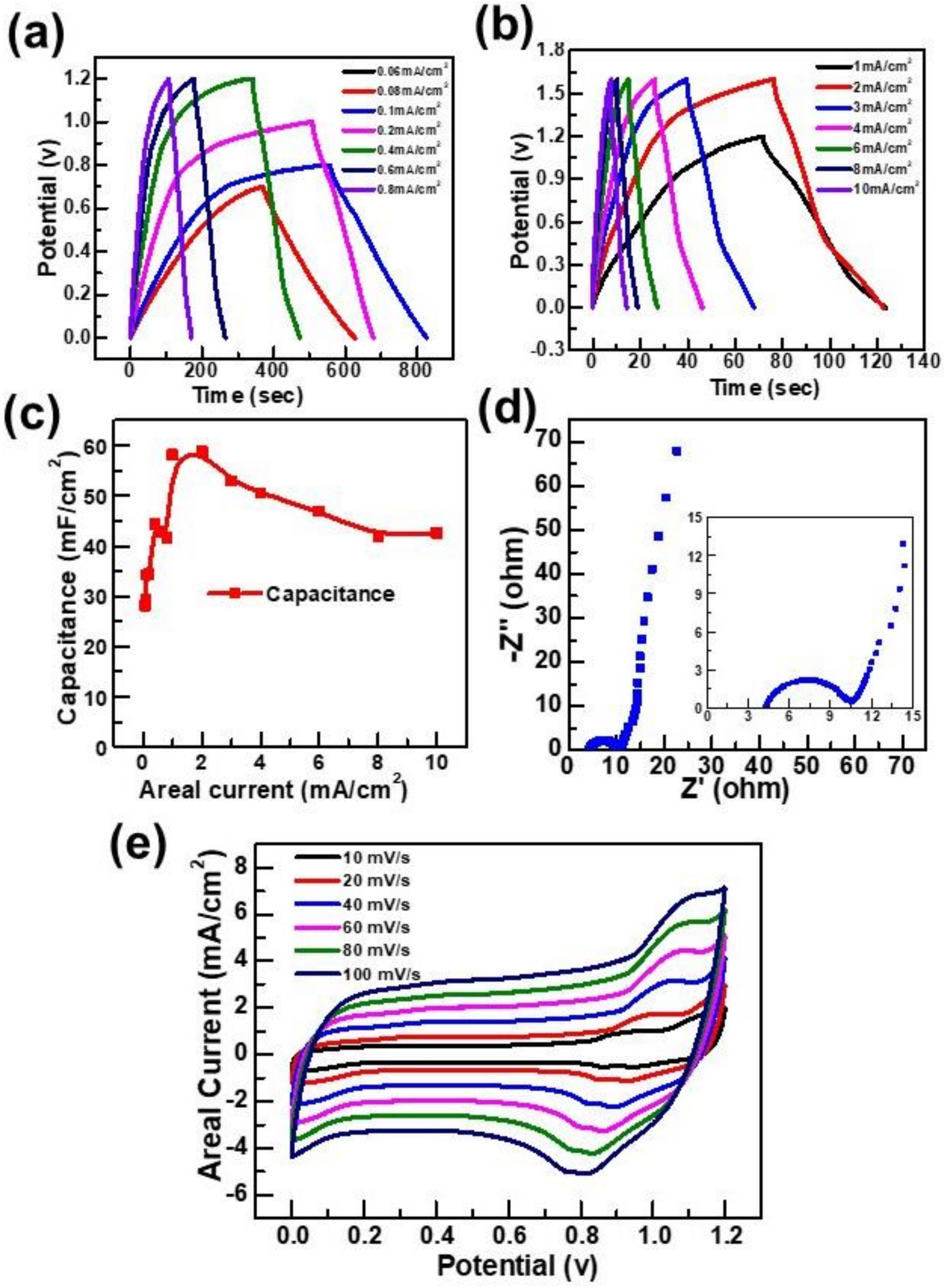

Figure S9. Electrochemical storage performance of NiO-A+CNTs//CNTs as an asymmetric full-cell: (a,b) CD curves, (c) Areal capacitance function, (d) Niquist plot of EIS, (e) CV curves.

Table S1. The maximum areal capacitance and the correspoding specific capacitance of all our half-cell electrodes.*

Electrode	Max. Areal Capacitance [mF/cm^2] @ Areal Current [mA/cm^2]	Mass Loading [mg/cm^2]	Max. Specific Capacitance [F/g] @ Current Density [A/g]
NiO-N	6.4 @ 0.2	1.5	4.3 @ 0.1
NiO-A	56.2 @ 0.2	1.4	40.2 @ 0.1
CNT	58.4 @ 0.8	1.9	30.7 @ 0.4
NiO-A+CNT	55.5 @ 2.0	2.9	19.1 @ 0.7

***Notes.** Our NiO-N and NiO-A electrodes have a mass loading of ~1.5 mg, while it is increased to ~3 mg after hybridization with CNT. The active materials of each of our samples were deposited on 1 cm^2 of carbon cloth substrate. The electrode mass loading was calculated by subtracting the mass of the uncoated substrate from the mass of the coated substrate. The table above clarifies that the maximum areal capacitances of NiO-A, CNT, and NiO-A+CNT electrodes are close to each other but at different areal currents, while their maximum specific (gravimetric) capacitances are separated by an order of magnitude. For more accurate comparisons of gravimetric capacitances, the mass of all compared electrodes should be identical. This condition was practically unattained using the electrospinning directly on the electrode substrate in our study. It has been experimentally proved and reported in the literature that the gravimetric capacitance of an electrode is usually changing by changing the mass loading even though the capacitance is divided by the mass. Many examples are well-investigated in these references:

- Chang L., Hang Hu, Y. *Breakthroughs in Designing Commercial-Level Mass-Loading Graphene Electrodes for Electrochemical Double-Layer Capacitors*, **Matter** 2019. 1 (3), 596-620. https://doi.org/10.1016/j.matt.2019.06.016
- Douard, C.; *et. al*. *Electrode Design for MnO_2-Based Aqueous Electrochemical Capacitors: Influence of Porosity and Mass Loading.* **Materials** 2021. 14, 2990. https://doi.org/10.3390/ma14112990
- Zhang, L., Gong, H. *A cheap and non-destructive approach to increase coverage/loading of hydrophilic hydroxide on hydrophobic carbon for lightweight and high-performance supercapacitors*. **Scientific Reports** 2016. 5, 18108. https://doi.org/10.1038/srep18108
